# Crosstalk between Stress Granules, Exosomes, Tumour Antigens, and Immune Cells: Significance for Cancer Immunity

**DOI:** 10.3390/vaccines8020172

**Published:** 2020-04-08

**Authors:** Vinoth Kumar Kothandan, Sangeetha Kothandan, Do Hee Kim, Youngro Byun, Yong-kyu Lee, In-Kyu Park, Seung Rim Hwang

**Affiliations:** 1 Department of Biomedical Sciences, Graduate School, Chosun University, 309 Pilmun-daero, Dong-gu, Gwangju 61452, Korea; 2 Department of Industrial Biotechnology, Bharath Institute of Higher Education and Research, Chennai 600073, India; 3 Department of Bioengineering, College of Engineering, Hanyang University, 222 Wangsimni-ro, Seongdong-gu, Seoul 04763, Korea; 4 College of Pharmacy, Seoul National University, 1 Gwanak-ro, Gwanak-gu, Seoul 08826, Korea; 5 Department of Molecular Medicine and Biopharmaceutical Sciences, Graduate School of Convergent Science and Technology, Seoul National University, 1 Gwanak-ro, Gwanak-gu, Seoul 08826, Korea; 6 Department of Chemical and Biological Engineering, Korea National University of Transportation, 50 Daehak-ro, Chungju, Chungbuk 27469, Korea; 7 Department of Biomedical Sciences, Chonnam National University Medical School, 322 Seoyang-ro, Hwasun 58128, Korea; 8 College of Pharmacy, Chosun University, 309 Pilmun-daero, Dong-gu, Gwangju 61452, Korea

**Keywords:** stress, exosome, cancer, immunotherapy, cancer/testis antigen

## Abstract

RNA granules and exosomes produced by tumour cells under various stresses in the microenvironment act as critical determinants of cell survival by promoting angiogenesis, cancer metastasis, chemoresistance, and immunosuppression. Meanwhile, developmental cancer/testis (CT) antigens that are normally sequestered in male germ cells of the testes, but which are overexpressed in malignant tumour cells, can function as tumour antigens triggering immune responses. As CT antigens are potential vaccine candidates for use in cancer immunotherapy, they could be targeted together with crosstalk between stress granules, exosomes, and immune cells for a synergistic effect. In this review, we describe the effects of exosomes and exosomal components presented to the recipient cells under different types of stresses on immune cells and cancer progression. Furthermore, we discuss their significance for cancer immunity, as well as the outlook for their future application.

## 1. Introduction

The tumour microenvironment consists of components, including cancer-associated fibroblasts (CAFs), immune-inflammatory cells, adipose cells, neuroendocrine cells, lymphatic vascular networks, and the extracellular matrix (ECM) [1]. Given the complex interplay between cells and the surrounding ECM, genetic or epigenetic alterations occur in cancers [2]. Among the hallmarks of cancer, the dysregulation of cellular signal transduction has significant characteristics, because proteins involved in signalling pathways, such as tyrosine kinase, GTPase, cytoplasmic serine–threonine kinase, and lipid kinase are genetically altered. In addition, cancer/testis (CT) antigens, which are generally expressed only in male germ cells, are aberrantly expressed in cancer and mediate the malignant phenotype [3]. Malignant tumour cells show continuous cell growth and division, resisting cell death and promoting angiogenesis, invasion, and metastasis [4]. They interact with heterotypic stromal cells by the release of extracellular vesicles (EVs) and the expression of signalling molecules for their survival and expansion [5]. 

When cells transformed into cancer cells are in an environment lacking oxygen and nutrients, RNA granules appear in the cytoplasm and regulate post-transcriptional gene expression [6]. In recent years, stress granules (SGs) have been identified as a promising target for the treatment of cancer, because the components involved in cancer cell physiology are often up-regulated in SGs and help the cancerous cells survive under the hostile microenvironment [7]. 

Under various stress conditions in the microenvironment, exosomes released from tumour cells promote progression of cancer metastasis [8,9]. Exosomes are nanovesicles released into the extracellular environment via the endosomal vesicle pathway by the fusion of a multivesicular body (MVB) with the cellular plasma membrane [10]. They contain a wide range of mRNAs, microRNAs (miRNAs), DNAs, and proteins, and their molecular composition varies according to the mechanism of formation, as well as the type or functional state of the donor cells [11]. Exosomes isolated from malignant effusions contain tumour antigens, such as human epidermal growth factor receptor 2 (HER2)/neu from ovarian cancer ascites and protein melan-A from melanoma patients [12,13]. In the tumour microenvironment, tumour-derived exosomes (TEXs) can interact not only with neoplastic cells but also with immune cells, thus modulating the anti-tumour immune response and affecting tumour progression [14]. Intercellular communication occurs in receptor–ligand interactions between membrane-bound proteins on exosomes and the corresponding receptors on the cell surface, followed by the activation of signalling, in cellular uptake of exosomes through phagocytosis, or in the direct fusion of exosomes with the cell membrane [15]. 

Exogenous anticancer therapies, as well as endogenous stressors in the tumour microenvironment, such as thermal stress, hypoxia, acidosis, and reactive oxygen species (ROS), trigger EV formation, resulting in the evolution of the microenvironment [16]. Exosomes carrying heat shock proteins (HSPs) from reticulocytes, peripheral blood mononuclear cells (PBMCs), B cells, and carcinoma cells have been reported to modulate immunological responses [17]. Tumour hypoxia, a stress condition where tumour cells are deprived of an oxygen supply, increases production and growth factor contents of exosomes, thereby promoting angiogenesis, invasion, and metastasis [18]. When disruption of EV membranes occurs in acidosis, vascular endothelial growth factor (VEGF) in the vesicles can be released and transferred to the recipient cells [19]. The contents of mRNA were also changed in the presence of oxidative stress, as demonstrated in mast cells, suggesting that the function of tumour-associated EVs can be altered by stress conditions [20]. The NKG2D transmembrane receptor on cytotoxic lymphocytes recognizes NKG2D ligands, which are induced during cellular stress. In malignant stages, however, cancer cells release NKG2D ligand-expressing exosomes as decoys for down-regulating the cognate receptor and impairing the NKG2D-mediated cytotoxic response [21].

While exosomes exposed to stress act as conveyors for tumour progression, they also exhibit delicate bystander effects [22]. Thus, utilizing these tumour-modulating effects of exosomes or customisation together with different immunotherapeutic approaches could be a promising way to treat cancer. One of the immunotherapeutic approaches is the activation of DNA damage response (DDR) for the clearance of senescent cancer cells by natural killer (NK) cells [23]. Targeting immunogenic cell death damage (danger)-associated molecular patterns (DAMPs) can be a strategy for strengthening the efficacy of cancer immunotherapy [24]. When intracellular biomolecules from the host are exposed outside the cells because of stress, tissue injury, or necrosis, they can be recognised as DAMPs through immune cell receptors [25]. Thus, DAMPs can initiate the innate immune system, including NK cells, and induce an adaptive T cell immune response by promoting the maturation of dendritic cells (DCs) and the presentation of antigens associated with dead tumour cells [26]. Various types of DAMPs, such as HSPs, high-mobility group box 1, DNA, and RNA have been reported to act as ligands of pattern recognition receptors and trigger signalling [27]. Immunogenic CT antigens overexpressed in malignancies have also been recognized as clinical targets for cancer immunotherapy [28]. 

**Figure 1:**
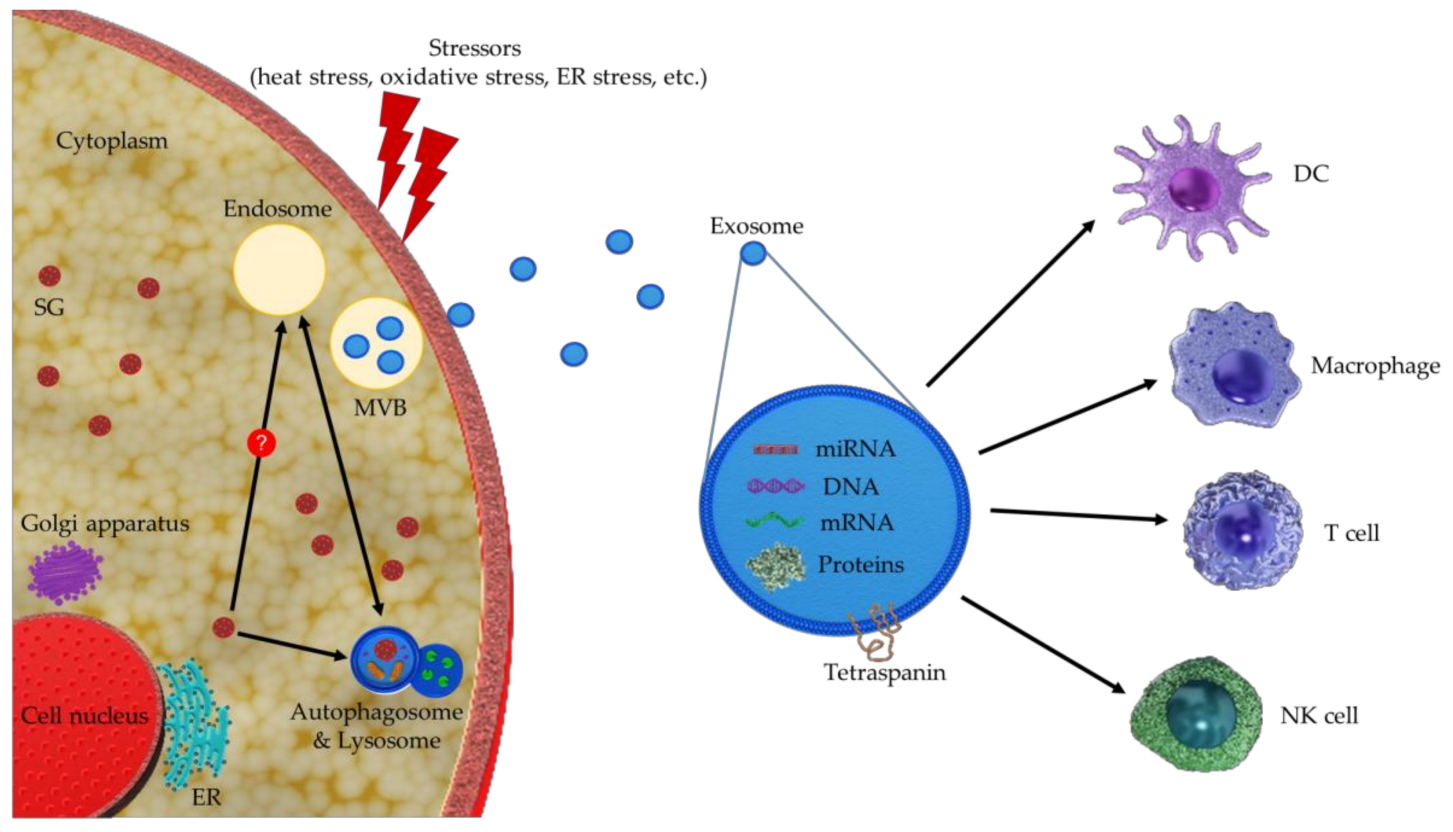
The outline of crosstalk between stress granules (SGs), exosomes, and immune cells. ER: endoplasmic reticulum; MVB: multivesicular body; miRNA: microRNA; DC: dendritic cell; NK: natural killer.

In this review, the influence of different stresses on RNA granules or exosomes and their interaction with immune cells, including DCs, macrophages, T cells, and NK cells will be described (Figure 1). The possible strategies for cancer immunotherapy, based on the components of SGs and exosomes together with CT antigens will also be discussed. The dysregulation of cellular signal transduction resulting from genetic alteration in proteins in the microenvironment would have significance for gametogenesis and cancer immunity.

## 2. SGs and RNA-Binding Proteins (RBPs) in the Tumour Microenvironment

**Scheme 1:**
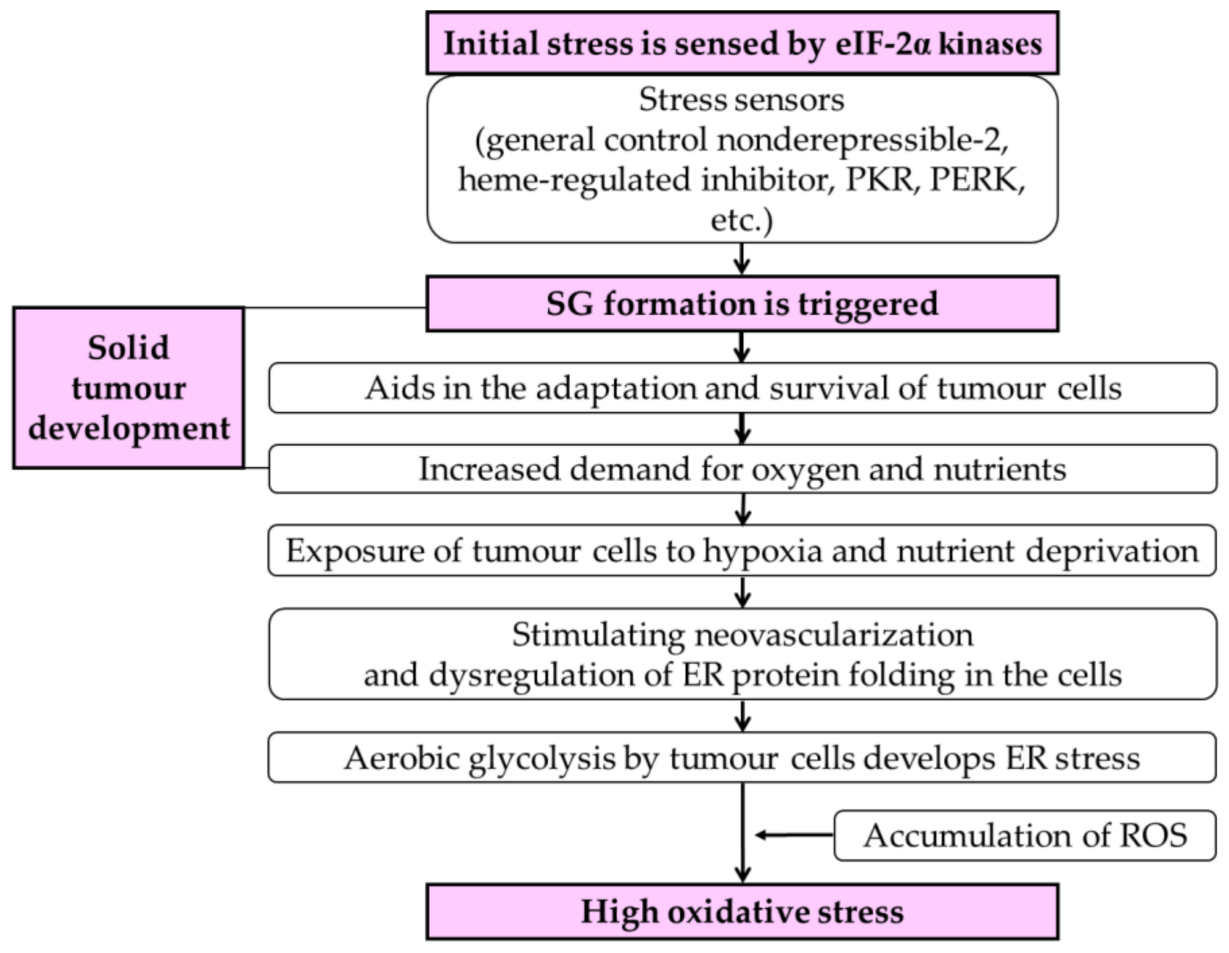
Schematic representation of the different stress pathways involved in the cancer microenvironment. eIF: eukaryotic initiation factor; PKR: protein kinase R; PERK: PKR-like endoplasmic reticulum kinase; SG: stress granule; ER: endoplasmic reticulum; ROS: reactive oxygen species.

SGs are the non-membranous, dense aggregation formed in the cytoplasm under different types of environmental stress [29]. After the initial stress in the cancer microenvironment is sensed by eukaryotic initiation factor (eIF)-2α kinases, such as general control nonderepressible-2, heme-regulated inhibitor, protein kinase R (PKR), and PKR-like endoplasmic reticulum kinase (PERK), SG formation is triggered [30]. Subsequently, it aids in the adaptation and survival of tumour cells, which are exposed to the increased demand for oxygen and nutrients during solid tumour development [31]. The exposure of tumour cells to an environment that induces suboptimal states, such as hypoxia and nutrient deprivation, stimulates neovascularisation and dysregulation of endoplasmic reticulum (ER) protein folding in the cells [31,32]. In addition to these conditions, aerobic glycolysis by tumour cells further develops ER stress and results in high oxidative stress, because of the accumulation of ROS (Scheme 1) [33]. 

As a result of phosphorylation of eIF-2 by stress, polysomes are disassembled and stalled messenger ribonucleoprotein particles arise in the cytoplasm [34]. These consist of translation initiation factors, 40s ribosomal subunits, and 48S complexes, as well as a subset of RNA-binding proteins (RBPs associated with mRNA as the main requirement for SG assembly, and subsequently, for the recruitment of other signalling molecules [35,36]. 

Critical roles of RBPs in SG assembly are evidenced by studies on the mutations of RNA recognition motifs and studies on the overexpression of RBPs, such as Ras GTPase-activating protein, SH3 domain binding protein (G3BP), and T-cell-restricted intracellular antigen 1 (TIA1) [37,38]. Further evidence has shown that the small-interfering RNA (siRNA)-mediated knockdown of TIA1, TIA1-like 1 (TIAL1 or TIAR), and G3BP1 significantly interferes with SG formation, despite the phosphorylation of eIF-2 [39]. The shuttling of RBPs in and out of SGs was confirmed by fluorescence recovery after photobleaching [40]. SGs are assembled by the shuttling of TIA1 with mRNA and equilibrate to polysomes dynamically [41]. Serine/arginine-rich splicing factor 3 protein also plays a role in SG assembly, because it is associated with 40s ribosomal subunits and the regulation of cytoplasmic post-transcriptional mRNA, as well as RNA splicing in the nucleus [42].

Y-box binding protein (YB)-1 with cold shock domain is also one of the protein components localised to SGs. It regulates SG formation by directly binding to the 5′ untranslated region (UTR) and activating translation of the mRNA encoding G3BP1 [43]. YB-1 expressed in the angiogenic endothelial cells of tumours serves as a transcriptional regulator, and silencing of YB-1 using siRNA significantly prevents growth factor-dependent cell growth [44]. YB-1 mRNA transcripts are regulated by the mammalian target of rapamycin (mTOR) complex 1, which contributes to tumour invasion and metastasis [45]. Increased levels of YB-1 alters the cellular localisation of membrane type 1 matrix metalloproteinase and activates the translation of mRNAs that mediate the epithelial–mesenchymal transition in breast carcinomas [46,47]. It is also correlated with poor prognosis or patient survival, as well as multidrug resistance-1 gene transcription [48,49]. This suggests that YB-1 is a potential target for cancer treatment. Additionally, YB-1 plays multifunctional roles, such as receptor activation, translation of oxidative phosphorylation mRNAs, and participation in the inflammatory processes [50,51,52]. 

It is worth noting that cytosolic YB-1 protein has been reported to be present in exosomes derived from the human embryonic kidney (HEK) 293 cells, and to recognise specific exosomal RNA motifs [53]. YB-1, which forms a complex with mRNA, can be transported out of the donor cell by YB-1-enriched vesicles, and can play a role in modulating target gene expression in the recipient cells [54,55].

The proteolytic cleavage of YB-1 can be controlled by the 20S proteasome [56]. It is important in YB-1 functions under stress, and thus, modulating proteolytic cleavage of YB-1 in the cancerous site could affect cell proliferation, invasion, and migration of immune cells. 

## 3. Influence of Exosomes Released under Stress on the Tumour Microenvironment

### 3.1. Exosomes Released from Cells Under Heat Stress

Exosomes include chaperone HSPs (e.g., Hsp60, Hsp70, Hsp84, and Hsp90), major histocompatibility complex (MHC) for antigen presentation and T cell stimulation, apoptosis-linked gene-2 interacting protein X (ALIX) or tumour susceptibility gene 101 for MVB formation, signal transduction proteins, adhesion molecules, cytoskeletal proteins, enzymes, and tetraspanins [57]. Stressors in the tumour microenvironment have been reported to change RNA or protein composition of exosomes and promote the release of exosomes [58]. Heat stress or hyperthermia activates the immune system and promotes granulocytic infiltration into the tumour site, which contributes to the control of tumour growth in vivo [59]. It might enrich intracellular chemokines in lipid rafts and increase the mobility of chemokines into exosomes via a lipid raft-dependent pathway [60]. Heat stress induces the expression of chemokines in TEXs, and chemotaxis may occur by the interaction of chemoattractive TEXs with DCs, activating T cells. Extracellular ATP release and calcium influx regulating vesicle transport are increased by heat treatment, which stimulates the release of exosomes from cells [61,62]. 

TEXs under heat stress have been reported to influence antitumour immunity in vitro and in vivo [63]. DCs incubated with TEXs under heat stress stimulated T cell proliferation and cytotoxic T lymphocyte (CTL) activity more efficiently than DCs incubated with untreated exosomes did in vitro. In heat-stressed exosomes from ascites of gastric adenocarcinoma patients, levels of Hsp60 and Hsp70 were higher than in stress-free exosomes [64]. Heat-stressed TEXs carrying carcinoembryonic antigens induced a tumour-specific CTL response and inhibited tumour progression in the colorectal adenocarcinoma mouse model more efficiently than TEXs without heat treatment [65]. HSPs upregulated by heat stress can be packed into exosomes and bind tumour antigens, leading to antigen cross presentation [66]. Hsp70 release from PBMCs under heat shock was reportedly dependent on exosomes rather than lipid rafts [67]. 

### 3.2. Exosomes Released from Cells under Oxidative Stress or Hypoxia

Oxidative stress induced by the imbalance between the level of ROS and detoxication ability triggers SG formation, and malignant tumour development is promoted by the increased production of certain ROS [68]. Hypoxia, a deficiency in the concentration of oxygen, also contributes to tumour aggressiveness and metastasis through hypoxia-inducible factor (HIF), leading to changes in metabolic pathways [69]. During oxidative stress or hypoxia triggered by tumour cells, the autophagy process in adjacent CAFs is induced with the aid of HIF-1α, and damaged proteins or organelles are removed [70,71]. EVs secreted from hypoxic tumour cells mediate the crosstalk between tumour cells and stromal cells in the tumour microenvironment [72,73]. Through the interaction, they can influence tumour angiogenesis, invasion, metastasis, and the immune system [74]. 

According to the donor cells, the content of exosomes under hypoxia vary in transcription factors, non-coding RNAs, mRNAs, fatty acids, and other proteins [75]. Upregulation of different miRNA expression has been reported in exosomes derived from various tumours under hypoxic stress [76]. Exosomes secreted from pancreatic cancer cells highly express miR-301a-3p under hypoxia and polarise anti-inflammatory M2 macrophages via phosphatase and tensin homolog (PTEN) downregulation and phosphoinositide 3-kinase (PI3K)-γ activation. M2 polarisation of macrophages incubated with hypoxic exosomes can be confirmed by M2 markers, such as cluster of differentiation (CD) 206, CD163, interleukin (IL)-10, transforming growth factor (TGF)-β, and arginase-1. Exosomal miR-301a-3p, which is dependent on HIF-1α and HIF-2α, leads to the enhanced invasion and metastasis of pancreatic cancer cells in vitro and in vivo [77]. Delivery of miR-25-3p by hypoxic exosomes also promotes IL-6 expression from macrophages; toll-like receptor (TLR)-dependent, nuclear factor, κ-light-chain-enhancer of activated B cells (NF-κB) activity; and migration of normoxic breast cancer cells [78].

The hypoxic condition in carcinoma tissue is correlated with proteins, which reduce cell adhesiveness and enhance invasion or migration of tumour cells [79]. Proteases, IL-1, VEGF, platelet-derived growth factor-BB, S100 calcium-binding protein A4, angiogenin, and granulocyte-macrophage colony-stimulating factor (GM-CSF) are secreted, according to quantitative analysis of the secretome from A431 tumour cells under hypoxia and reoxygenation [80]. More than 40% of the secretomes from hypoxic carcinoma cells were found to be exosome-associated cytosolic proteins, including tetraspanins and ALIX, and to have the potential for activating the proteolytic system, angiogenesis, and metastasis. This suggests that exosomal cargo protein contents changed under hypoxic stress affect the transcriptome of recipient cells and enable tumour cells to overcome the hostile microenvironment. Exosomes derived from hypoxic glioma cells included neovascularisation-regulating proteins and increased the tube formation or branching intervals in endothelial progenitor cells, possibly via the upregulation of small nucleolar RNA and downregulation of the voltage-gated potassium channel gene [81]. 

Hypoxic tumour cells secrete exosomes carrying TGF-β, resulting in immunosuppression through the accumulation of regulatory T (Treg) cells and activation of CAFs [82]. TEXs containing NKG2D ligands under hypoxic stress impair the cytolytic functions of NK cells via the downregulation of NKG2D receptors [83]. Moreover, chemoattractants, such as colony-stimulating factor (CSF)-1 and ferritin heavy/light chains, are enriched in exosomes secreted from hypoxic tumour cells, which contribute to monocyte recruitment and macrophage differentiation [84,85]. The differentiation of monocytic cells to M0-like macrophages and their polarisation to M2 macrophages were supported by the exosomal cargo from human melanoma, epidermoid carcinoma, and lung cancer cells under hypoxic stress [85]. The treatment of hypoxic exosomes also induced stem cell differentiation, which was confirmed by F4/80^+^CD135^high^ positive cell populations in bone marrow-derived macrophages (BMMs). In addition, let-7a miRNA enriched in hypoxic TEXs inhibited the insulin-protein kinase B (AKT)-mTOR signalling pathway, leading to enhanced mitochondrial oxidative phosphorylation of BMMs. 

Overall, the detailed analysis of hypoxic tumour cells and TEXs in cancer biology have provided positive signs for the angiogenesis, invasion, metastasis, and progression of cancer through the abnormal expression of specific proteins or RNAs, but have a negative effect on anticancer immunity. 

### 3.3. Exosomes Released from Cells under ER Stress

When the integrity of the ER in tumour cells is disturbed by exposure to an environment lacking oxygen and nutrients, accumulation of misfolded proteins in the ER lumen, an imbalance in calcium homeostasis, and dysregulation of lipid metabolism occur, leading to ER stress [86]. In response to ER stress, an adaptive intracellular signalling pathway, known as the unfolded protein response (UPR), is activated and controls cell fate [87]. The UPR in eukaryotic cells has the following functions: (1) attenuation of protein translation to reduce further translational loads on the ER, (2) the upregulation of molecular chaperone genes involved in protein folding and degradation, and (3) ER-associated degradation clears misfolded proteins by retro-translocating these proteins from the ER lumen into the cytosol through the ubiquitin–proteasome system [88]. 

There is evidence that control of ER proteostasis via UPR may affect cancer progression [89]. Proteostasis regulated by transmembrane receptor proteins, such as inositol-requiring enzyme (IRE) 1, activating transcription factor 6, and PERK, is linked to immune responses [90]. ER stress can be transferred between cells during the culture of macrophages in conditioned medium from ER-stressed tumour cells with the upregulation of ER stress response genes, such as Grp78, as well as splicing of the mRNA encoding X-box binding protein (XBP) 1, which expands proinflammatory response and activates Wnt signalling [91].

Attenuation of anticancer immune surveillance caused by ER stress might be related to exosomes. Increased exosomal miR-23a-3p levels in ER-stressed hepatocellular carcinoma tissues were correlated with the increased expression of programmed cell death (PD)-ligand 1 (L1) in macrophages, and inversely correlated with patient survival [92]. Severe ER stress in choriocarcinoma cells stimulated the secretion of exosomes carrying DAMP molecules, which might contribute to the inflammatory response [93]. ER stress enhances MVB formation and the release of exosomes, which are related to IRE1- and PERK-mediated pathways [94]. In IRE1α-overexpressed HEK293T cells, the spliced form of XBP1 mRNA was distinctly incorporated into exosomes [95]. Treatment with TEXs increased the expression of ER stress-related genes in normal endothelial cells, disrupted vascular integrity, and promoted tumour metastasis [96]. 

Overall, as mentioned above, stress and exosomes derived from tumours have effects on different immune or myeloid components (Table 1). 

## 4. Cancer Immunotherapy Based on SG Components or Exosomes for Clinical Application

Recently, the paradigm of anticancer drug development has shifted to cancer immunotherapy to overcome the limitations of chemotherapy and targeted therapy. Cancer immunotherapy is based primarily on strategies for attacking cancer cells by activating T lymphocytes. Immune checkpoint blockers, including anti-cytotoxic T lymphocyte-associated protein (CTLA)-4 and anti-PD1/PD-L1, have been approved by the U.S. Food and Drug Administration (FDA) and are used for the treatment of melanoma and non-small-cell lung carcinoma (NSCLC) [97]. 

As exosomes carry informative proteins, RNA, and DNA from the donor cells to the recipient cells, they affect not only the microenvironment around the cells, but also pathophysiological conditions. The unique exosomal cargo that affects the elimination of obsolete proteins and recruitment, differentiation, or polarisation of immune cells has been recognised as an appealing target for novel cancer immunotherapy [98]. Owing to the antigen-presenting characters of DCs and the biocompatibility of exosomes, clinical trials using autologous DC-derived exosomes (DEXs) in metastatic melanoma and NSCLC patients have already been conducted [99,100] (Table 2). It has been suggested that injections of DEXs harbouring MHC/tumour peptide complexes would mediate tumour growth retardation with MHC-restricted antigens presenting CD4^+^ and CD8^+^ T cell responses. DEXs isolated from bone marrow- or monocyte-derived DCs possess NKG2D ligands and IL-15Rα, which trigger the activation and proliferation of NK cells in patients [101]. Cellular stress and tumourigenesis activate pathways for regulating the expression of NKG2D ligands excreted on exosomes, which activate the DDR to repair DNA lesions. To boost NK and CTL responses in NSCLC patients after first-line chemotherapy, exosomes derived from IFN-γ-matured DCs loaded with MHC-restricted cancer antigens were tested in a recent phase II clinical trial [102].

TEXs enable tumour cells to communicate with and reprogram the recipient stromal cells, immune cells, or bone marrow-derived cells [103]. Despite their delivery of tumour antigens, HSPs, and MHCs, TEXs show somewhat suppressive effects on cancer immunity by inducing apoptosis of CD8^+^ T cells, inhibition of monocyte differentiation, suppression of NK cell functions, and the stimulation of regulatory immune cells, such as myeloid-derived suppressor cells (MDSCs) and Treg cells [104]. To potentiate T cell activation and the antitumour immunity of TEXs, researchers have attempted to isolate TEXs from heat-stressed tumour cells and modify them with superantigens, cytokines, or HSP70 [105,106,107]. Malignant effusions or autologous ascites from patients were clinically tested as the source for large amounts of exosomes and carcinoembryonic antigens, in combination with the adjuvant GM-CSF, which could beneficially induce the antitumour CTL response without significant toxicities [108]. Mesenchymal stem cell-derived exosomes that show potential in other disease models are also a candidate for cancer immunotherapy [109].

To enhance the efficacy of exosome-based therapeutics, researchers have tried adding or combining exosomal contents and other functional ingredients together [110]. Exosomes engineered with the variant of signal regulatory protein α (SIRPα) antagonise the interaction between CD47 and tumour cells and between SIRPα and macrophages, and increase tumour phagocytosis. Treatment of tumour-bearing mice with exosomes harbouring SIRPα variants have led to intensive infiltration of CD8^+^ T cells in the tumour microenvironment, as well as subsequent inhibition of tumour growth [111]. Exosomes derived from hyaluronidase-anchored HEK293T cells, in combination with PD-L1 blocker, exhibited the stimulation of DCs and an antitumour CD8^+^ T cell response in mice [112].

Meanwhile, SG formation in tumour cells under stress conditions favours tumour cell survival and confers resistance to anticancer drugs. In some cases, solid tumours may show resistance to Bortezomib, a proteasome inhibitor for the treatment of mantle cell myeloma and multiple myeloma, which is thought to be related to the activation of the stress response [113]. There is an important relationship between selective mRNA translation, stress adaptation, SGs, and tumour progression. Blocking expression of SG-specific proteins, such as YB-1 and G3BP1, significantly reduces tumour progression and metastasis. Intracellular microtubules transport SGs carrying immune checkpoint mRNA to regulate mRNA translation, and T lymphocytes rapidly increase PD1 mRNA expression as soon as T cell receptors are activated [114]. 

## 5. Exosomal Expression Linked to Cancer Progression: ONCOGENIC miRNAs (onco-miRs) and Tumour Suppressor miRNAs Together with CT Antigens

RNA analysis revealed that exosomes contain short, non-coding miRNAs with 19−22 nucleotides, as well as mRNAs [115]. The exosomal miRNA cargo exchanged between cells can play a role in drug resistance and the diagnosis of cancer [116]. The miR-155 upregulated in cancer stem cells or chemoresistant cells was transferred to the sensitive recipient cells by exosomes, which mediated breast cancer chemoresistance [117]. Oncogenic miRNAs (onco-miRs) overexpressed in adenocarcinomas, such as miR-223-5p, miR-223-3p, miR-483-5p, miR-409-3p, miR-196-5p, miR-192-5p, miR-146a-5p, and miR-126-5p, were also identified in exosomes isolated from the serum of adenocarcinoma patients [118]. 

Moreover, exosomal miRNAs mediate the ECM crosstalk between cancer cells and CAFs, and influence the activation of CAFs [119]. Delivery of miR-155 to the recipient cells by pancreatic, ductal, adenocarcinoma-derived exosomes downregulated the stress-induced p53 target gene, which activated the conversion of normal fibroblasts to CAFs [120]. The miR-1247-3p in hepatocellular carcinoma cell (HCC)-derived exosomes targets β-1,4-galactosyltransferases III in fibroblasts and activates β1-integrin-NF-κB signalling, leading to liver cancer progression and lung metastasis [121]. Exosomal miR-21 and miR-29a in non-small-cell lung carcinoma (NSCLC) can be bound to TLRs, which mediate the prometastatic inflammatory response [122]. According to another study, pancreatic cancer-secreted exosomes transfer miR-212-3p to DCs and inhibits the expression of the transcription factor for MHC II, resulting in immune tolerance [123]. The suppressed proliferation of the recipient PBMCs or macrophages, as well as stimulated tumour cell proliferation, might be caused by the transfer of miR-125a by TEXs [124]. 

Additionally, miR-584 enriched in HCC-derived exosomes promotes HCC progress by downregulating TGF-β-activated kinase 1, which controls the cell death response to environmental stress [125]. In lung adenocarcinoma cells, miR-210 is up-regulated by the tissue inhibitor of metalloproteinases-1 along the PI3K/AKT/HIF-1-dependent pathway, and miR-210-accumulated exosomes promote angiogenesis in vitro and in vivo [126]. Studies on exosomal miRNAs under different stress conditions, however, are quite insufficient to date. Further analysis of exosomal gene expression in tumour cells under stress conditions could aid in the identification of the specific targets for cancer immunotherapy. On the other hand, in more advanced metastatic colon cancer, circulating exosomes rather than tumour tissue are enriched with miR-193a, one of the tumour suppressor miRNAs [127]. The tumour-suppressing miR-200 family released into exosomes negatively regulates zinc finger E-box-binding homeobox 1 protein, which contributes to metastasis [128]. 

Interestingly, several CT antigens are also expressed in exosomes as well as the corresponding tumours. Melanoma-associated antigen (MAGE)-B4 showed higher gene expression in urinary exosomes from bladder cancer patients than that in normal control samples [129]. Testis-specific lactate dehydrogenase C4 was upregulated in serumal exosomes from breast cancer patients [130]. CT antigen SPANXB1 was exclusively expressed in circulating small EVs and promoted the metastasis of triple-negative breast cancer [131]. In other words, expression of CT antigens correlates with tumour progression, and tumour-expressed CT antigens can be combined with exosomes for antigenic presentation. Support of cell-division cycle processes and suppression of apoptosis signaling cascades, as well as the regulation of transcriptional activity involved in gametogenesis via CT antigens, affects tumour growth and metastasis. Although CT antigens on male germline cells, which do not express MHC molecules, cannot be recognized by T cells, DNA demethylation in tumours allows attack by lymphocytes [132,133]. As immunogenic CT antigens are selectively expressed to tumor tissues and exosomes, they have potential as antigen-specific cancer vaccines or diagnostic markers. Clinical trials using CT antigens, such as MAGE (-A1, A3, A4, and A10) and NY-ESO-1, have been conducted (Table 3). 

## 6. Conclusions and Future Perspectives

As seen from the tumour–host crosstalk and cell-to-cell communication at the primary tumour site and distant sites, a multicentric adaptive therapeutic approach is needed for the efficient treatment of cancer [134]. The careful examination of signalling pathways presents an intertwining network involving the partial recruitment of immune cells at each stage, either to check cancer progression or sometimes to escape cancer progression. Although few cancers share specific proteins in common at the beginning, the phenotypic heterogeneity exhibited by different cancer cells and among their own populations clearly makes the current targeting techniques for one specific target doubtful. 

Hence, the extension of knowledge regarding exosomal components and crosstalk with immune cells under stress in the tumour microenvironment enables us to pool and select candidates for cancer immunotherapy, with the hope of overcoming the feedback mechanisms involved in immunological evasion for cancer progression. Furthermore, novel therapeutic targets, which show higher expression in both testicular germ cells and cancer cells than expression in other normal tissues, and which are closely associated with the pathogenesis of cancer or cell cycle maintenance, can be explored for their synergistic anticancer effect [135].

## Figures and Tables

**Table 1:** Effects of stress and exosomes derived from tumours on different immune or myeloid components.

Stress/Exosome	Immune/Myeloid Component	Effects
Oxidative stress or hypoxia	Tumour-associated macrophage (TAM)	Infiltration of macrophages, differentiation of TAM into the M2 phenotype, protumoral effects
ER stress	Tumour-associated DC	XBP1 activation, impairing antigen-presenting capacity, cancer progression
Exosome isolated from heat-stressed tumour	DC, T cell	Chemoattracting DC and T cell, antitumour immune response, inhibiting tumour growth
Exosomes derived from hypoxic cancer	Macrophage	M2-type polarization, proangiogenic factor expression, cancer progression
TEX in the tumour microenvironment	Myeloid-derived suppressor cell (MDSC)	Expansion of MDSC, immunosuppression

TAM: tumour-associated macrophage; ER: endoplasmic reticulum; DC: dendritic cell; XBP: X-box binding protein; TEX: tumour-derived exosome; MDSC: myeloid-derived suppressor cell.

**Table 2:** Cancer immunotherapy using SG components or exosomes in clinical trials.

Cancer	SG Components or Exosomes	Clinical Trial Phase	Dosage Regimen	Reference
HER2- negative stage III–IV breast cancer	CD105/YB-1/SOX2/ CDH3/MDM2- polyepitope plasmid DNA vaccine with recombinant human GM-CSF	Phase I	Every 28 days for 3 months and booster vaccines at 6 and 12 months	ClinicalTrials.gov Identifier: NCT02157051 (https://clinicaltrials.gov/ct2/show/NCT02157051)
Metastatic melanoma	Autologous PBMC-based DEXs loaded with MAGEs	Phase I	Four vaccinations at weekly intervals	Escudier et al. [99]
Advanced NSCLC	Autologous PBMC-based DEXs pulsed with antigenic peptides	Phase I	Four vaccinations at weekly intervals	Morse et al. [100]
Colorectal cancer	Exosomes derived from autologous ascites combined with GM-CSF	Phase I	Four vaccinations at weekly intervals	Dai et al. [108]
Advanced NSCLC	IFN-γ-matured DEXs loaded with MHC-restricted cancer antigens	Phase II	Four vaccinations at one-week intervals after cyclophosphamide, followed by six vaccinations at two-week intervals	Besse et al. [102]

SG: stress granule; HER2: human epidermal growth factor receptor 2; YB: Y-box binding protein; GM-CSF: granulocyte-macrophage colony-stimulating factor; PBMC: peripheral blood mononuclear cell; DEX: dendritic cell-derived exosome; MAGE: melanoma-associated antigen; NSCLC: non-small-cell lung carcinoma; IFN: interferon; MHC: major histocompatibility complex.

**Table 3:** Cancer immunotherapy targeting CT antigens in clinical trials.

Cancer	Detailed Description	Clinical Trial Phase	References (ClinicalTrials.gov Identifier)
Multiple myeloma	MAGE-A3 and NY-ESO-1 (peptide vaccination) combined with chemotherapy and auto transplantation	Phase II, Phase III	NCT00090493 (https://clinicaltrials.gov/ct2/show/NCT00090493)
Stage III or IV melanoma	Melanoma antigen peptides (including NY-ESO-1b analog and MAGE-A10) and Montanide adjuvant	Phase I	NCT00112242 (https://clinicaltrials.gov/ct2/show/NCT00112242)
Metastatic melanoma	Autologous DCs transfected with tumour antigen (including MAGE-A3) RNA and proteasome siRNA	Phase I	NCT00672542 (https://clinicaltrials.gov/ct2/show/NCT00672542)
Metastatic melanoma and renal cell cancer	Chemotherapy followed by anti-NY-ESO-1 T-cell receptor gene engineered lymphocytes	Phase II	NCT00670748 (https://clinicaltrials.gov/ct2/show/NCT00670748)
Melanoma	MAGE-A3 plusGSK2132231A (antigen-specific cancer immunotherapy)	Phase III(failed)	NCT00796445 (https://clinicaltrials.gov/ct2/show/NCT00796445)
NY-ESO-1- expressing tumours	CpG 7909/ Montanide in combination with NY-ESO-1 peptide or protein	Phase I	NCT00819806 (https://clinicaltrials.gov/ct2/show/NCT00819806)
Melanoma (expressing NY-ESO-1)	Topical Resiquimod as an adjuvant for NY-ESO-1 protein, plus Montanide vaccination	Phase I	NCT00821652 (https://clinicaltrials.gov/ct2/show/NCT00821652)
Melanoma	NY-ESO-1 protein and TLR3 agonist adjuvant	Phase I, Phase II	NCT01079741 (https://clinicaltrials.gov/ct2/show/NCT01079741)
NSCLC	Chemotherapy and DEXs (peptides like MAGEs and NY-ESO-1 pulsed onto DC)	Phase II	NCT01159288 (https://clinicaltrials.gov/ct2/show/NCT01159288)
Neuroblastoma and sarcoma	Mature DC pulsed with peptides derived from NY-ESO-1, MAGE-A1, and MAGE-A3, preceded by chemotherapy	Phase I	NCT01241162 (https://clinicaltrials.gov/ct2/show/NCT01241162)
Metastatic cutaneous melanoma	NY-ESO-1 plus GSK2241658A (antigen-specific cancer immunotherapy)	Phase I	NCT01213472 (https://clinicaltrials.gov/ct2/show/NCT01213472)
Myeloma	MAGE-A3 immunization with adjuvant Hiltonol plus activated autologous T cells	Phase II	NCT01245673 (https://clinicaltrials.gov/ct2/show/NCT01245673)
Hodgkin’s or non-Hodgkin’s lymphoma	CTL primed against tumour-associated antigens, including MAGE-A4 and NY-ESO-1	Phase I(ongoing)	NCT01333046 (https://clinicaltrials.gov/ct2/show/NCT01333046)
Synovial sarcoma	Genetically engineered NY-ESO-1-specific T cells with chemotherapy	Phase I	NCT01343043 (https://clinicaltrials.gov/ct2/show/NCT01343043)
Multiple myeloma	Autologous T cells expressing high affinity, TCR-specific for NY-ESO-1	Phase II	NCT01352286 (https://clinicaltrials.gov/ct2/show/NCT01352286)
Stage IIB–IV resected melanoma	Recombinant MAGE-A3 protein combined with immunological adjuvant system	Early Phase I	NCT01425749 (https://clinicaltrials.gov/ct2/show/NCT01425749)
NY-ESO-1- expressing solid tumours	CD205-NY-ESO-1 fusion protein with or without sirolimus	Phase I	NCT01522820 (https://clinicaltrials.gov/ct2/show/NCT01522820)

CT: cancer/testis; MAGE: melanoma-associated antigen; DC: dendritic cell; TLR: toll-like receptor; NSCLC: non-small-cell lung carcinoma; DEX: dendritic cell-derived exosome; CTL: cytotoxic T lymphocyte; CD: cluster of differentiation.
